# Tinnitus: Clinical Insights in Its Pathophysiology-A Perspective

**DOI:** 10.1007/s10162-024-00939-0

**Published:** 2024-03-26

**Authors:** Berthold Langguth, Dirk de Ridder, Winfried Schlee, Tobias Kleinjung

**Affiliations:** 1 Department of Psychiatry and Psychotherapy, University of Regensburg, Regensburg, Germany; 2 Interdisciplinary Tinnitus Clinic, University of Regensburg, Regensburg, Germany; 3 Section of Neurosurgery, Department of Surgical Sciences, Dunedin School of Medicine, University of Otago, Dunedin, New Zealand; 4 Eastern Switzerland University of Applied Sciences, St. Gallen, Switzerland; 5 Department of Otorhinolaryngology, University Hospital Zurich, University of Zurich, Zurich, Switzerland

**Keywords:** Tinnitus, Aetiology, Comorbidity, Pathophysiology, Treatment, Heterogeneity

## Abstract

Tinnitus, the perception of sound without a corresponding external sound source, and tinnitus disorder, which is tinnitus with associated suffering, present a multifaceted clinical challenge due to its heterogeneity and its incompletely understood pathophysiology and especially due to the limited therapeutic options. In this narrative review, we give an overview on various clinical aspects of tinnitus including its heterogeneity, contributing factors, comorbidities and therapeutic pathways with a specific emphasis on the implications for its pathophysiology and future research directions. Tinnitus exhibits high perceptual variability between affected individuals (heterogeneity) and within affected individuals (temporal variability). Hearing loss emerges as predominant risk factor and the perceived pitch corresponds to areas of hearing loss, supporting the compensatory response theory. Whereas most people who have tinnitus can live a normal life, in 10–20% tinnitus interferes severely with quality of life. These patients suffer frequently from comorbidities such as anxiety, depression or insomnia, acting as both risk factors and consequences. Accordingly, neuroimaging studies demonstrate shared brain networks between tinnitus and stress-related disorders shedding light on the intricate interplay of mental health and tinnitus. The challenge lies in deciphering causative relationships and shared pathophysiological mechanisms. Stress, external sounds, time of day, head movements, distraction, and sleep quality can impact tinnitus perception. Understanding these factors provides insights into the interplay with autonomic, sensory, motor, and cognitive processes. Counselling and cognitive-behavioural therapy demonstrate efficacy in reducing suffering, supporting the involvement of stress and anxiety-related networks. Hearing improvement, especially through cochlear implants, reduces tinnitus and thus indirectly validates the compensatory nature of tinnitus. Brain stimulation techniques can modulate the suffering of tinnitus, presumably by alteration of stress-related brain networks. Continued research is crucial for unravelling the complexities of tinnitus. Progress in management hinges on decoding diverse manifestations, identifying treatment-responsive subtypes, and advancing targeted therapeutic approaches.

## Introduction

### The Relevance of Clinical Observations

Historically, progress in medicine usually begins with a comprehensive description of clinical symptoms and their definition. In a next step, commonly occurring symptoms are grouped into syndromes and pathophysiological models are developed based on their features. This process is supported by histological and anatomical research as well as by imaging, laboratory and genetic studies, to identify structural correlates of the clinical syndromes. Pathophysiological models form the basis for the development of therapeutic approaches. In many cases, therapeutic effects have been discovered by chance, e.g. in the case of neuroleptics. These serendipitous discoveries provide further hints for understanding the pathophysiology of a particular disorder. Thus, from a historical perspective, clinical observations form the basis for the development of pathophysiological models which in turn serve for a better understanding of a disease or a disorder.

### Pathophysiological Models of Tinnitus

With respect to tinnitus, it is frequently stated that its pathophysiology is incompletely understood. Indeed, there exist many pathophysiological models of tinnitus (which are not mutually exclusive), but none of them can comprehensively explain all relevant clinical aspects of tinnitus. The *Peripheral Model* involves dysfunction in the auditory periphery, such as damage to the cochlea or auditory nerve. This can lead to abnormal spontaneous neural activity, interpreted by the brain as sound [1]. The *Central Model* focuses on changes in central auditory pathways, triggered by reduced auditory input. These changes may involve neurotransmitter imbalances as well as increased neuronal activity and synchrony [2]. In addition to altered activity in the brain’s auditory processing centres, there are activity and connectivity alterations in non-auditory networks, particularly in salience—emotion processing—and executive networks [3, 4, 6]. The *Gating Model* combines these models by postulating that tinnitus emerges, when there is increased activity in central auditory pathways together with a frontostriatal inhibitory deficit, which would normally prevent the signal to reach conscious perception [5]. The *Somatosensory Model* is focusing on abnormal interactions between the auditory and somatosensory systems which finally result in increased neuronal activity in central auditory pathways [7]. Most recently also an *Inflammatory Model* [8] has been proposed as an explanation for the emergence of tinnitus. In addition to these pathophysiological models, there exist also *Psychological Models* [9] and *Models based on Perception Theory* [10, 11].

### Heterogeneity of Tinnitus

The question arises why a condition such as tinnitus with a high prevalence [12] and high socioeconomic relevance [13] is still a mystery today. The search for explanations for this unsatisfactory situation almost always leads to the fact that tinnitus is a far from homogeneous clinical entity [14, 15]. Etiologic factors and triggers vary from patient to patient, but perceptual aspects, comorbidities, burden, modifying factors and response to therapeutic interventions also vary widely. A generally valid pathophysiological model must take this variability into account or at least explain the characteristics of clearly defined, distinct subtypes. Since such pathophysiological models do not yet exist, it also remains a matter of debate whether there is a “final common pathophysiological pathway” or, in other words, pathophysiological alterations that can be found in every single tinnitus patient, or whether there are different forms of tinnitus with different pathophysiological changes that may not overlap at all. For both scenarios, there exist examples in medicine. Schizophrenia is a disorder, which varies highly in its clinical course and its symptomatology. However, pharmacological blockade of dopamine receptors can reduce clinical symptoms in almost all cases, indicating that the dysregulation of dopaminergic pathways represents a common pathway that is relevant for all forms of schizophrenia. In contrary in headache differentiation in subtypes has been essential for successful treatment. The International Classification of Headache Disorders (ICHD) lists more than 200 subtypes and there exist specific treatments for these various subtypes. Migraine, tension type headache, cluster headache or trigeminal neuralgia—just to name a few—can be best treated by completely different treatment regimes.

In this perspective article, we will discuss various clinical aspects of tinnitus and the insights into pathophysiological mechanisms that we can derive from these clinical phenomena. We will also discuss those clinical aspects, which are currently still incompletely understood as these latter ones provide hints in which direction future research should be directed.

## Etiological Factors

According to a recent systematic review [16], hearing loss, occupational noise exposure, otitis media, ototoxic medication and depression were identified as main risk factors for the development of tinnitus.

### Hearing Loss

These findings confirm that deprivation of auditory input is the most relevant risk factor for the development of tinnitus [1]. This notion is also supported by the finding that the laterality and frequency of tinnitus typically correspond with the hearing loss [17, 18]. For example, a patient with a left-sided hearing loss at a frequency of 4 kHz typically perceives his tinnitus as a 4 kHz tone on the left side. However, not everybody with hearing loss develops tinnitus and not all patients with tinnitus experience hearing loss. It is controversial whether tinnitus patients with normal hearing thresholds really have completely normal hearing or whether they have a form of hearing impairment that remains undetected by the standard audiogram that samples only 8 frequencies out of the human auditory spectrum, which ranges from about 20 Hz till 20 kHz. Studies on such subjects have shown that they may have hearing loss in the ultra-high frequency range, which is not routinely sampled [19, 20], hearing loss between tested frequencies [21] or damage to high threshold auditory nerve fibres [22]. An alternative explanation for hearing loss without tinnitus is a delay between the onset of hearing loss and the onset of tinnitus. In this case, cross-sectional studies will always detect patients with hearing loss—but without tinnitus. We will need large longitudinal data sets and to follow up patients with hearing loss to learn how many of them will develop tinnitus—and after how many years this happens. Furthermore, the lacking one-to-one correlation between hearing loss and tinnitus points to additional factors, which might play a role.

### The Somatosensory System

There are several lines of evidence that indicate the involvement of the somatosensory system. First, the majority of tinnitus patients can modulate their tinnitus by moving their head [23]. Second, there is an increased tinnitus prevalence among patients with temporomandibular joint disorder, and in a subset of patients, the onset of tinnitus is triggered by neck trauma, such as whiplash injury [24]. Animal research has contributed to the identification of the pathophysiological mechanisms by demonstrating that altered input from the somatosensory system can influence activity in central auditory pathways via C2 and trigeminal afferents, which interact with auditory input at the dorsal cochlear nucleus [7].

### Multimodal Interactions

The somatosensory modulation of tinnitus perception and the co-occurrence of tinnitus with different kinds of pain [25–27] suggest that some form of multimodal processing may be required for tinnitus generation and or tinnitus maintenance [11]. In other words, “*When I see a bird that walks like a duck and swims like a duck and quacks like a duck, I call that bird a duck*” [28]. This may imply that missing auditory input may be compensated for by somatosensory inputs based on the duck test mechanism [11]. Intriguingly, not only the somatosensory system may be involved in tinnitus. Visual snow, the visual analogue of tinnitus [29], is comorbid with tinnitus [30], and cataract is a risk factor for tinnitus [31]. This suggests that possibly not only the somatosensory system is involved in tinnitus generation but in some patients the visual system may influence it. And similarly, the vestibular system may be involved. Indeed, in 1000 patients presenting at an otology clinic, tinnitus was present in 70%, imbalance in about 25%, otalgia and aural fullness in about 20%, with more than one symptom occurring in 25% of patients [32, 33]. The comorbidity of tinnitus and vestibular systems is most outspoken in Meniere’s disease, in which tinnitus and vertigo are part of the diagnostic criteria [34, 35].

### Depression and Anxiety

Depression is another risk factor for tinnitus [36] and even more so in tinnitus with comorbid symptoms. In Meniere’s disease, a meta-analysis has shown that the prevalence of depression is close to a staggering 50% [37]. Similarly, anxiety is associated with tinnitus [38]. This suggests an overlap of brain networks associated with tinnitus, anxiety and depression or that tinnitus and comorbid symptoms can trigger activation of depression networks [6, 39–41]. Presumably, these are the hippocampal–cortical memory system [11], the default mode network, the frontoparietal control system and brain areas for salience and emotion processing [3, 42]. The involvement of these structures might reflect the conscious perception of tinnitus, the attention to it, its salience and the associated distress [4, 42]; in other words, the unified tinnitus percept may result from multiple, parallel, overlapping and interacting networks [6]. If this model is correct then every aspect of the unified tinnitus percept may be related to one network, e.g. loudness to auditory-memory-salience network [43–45], arousal/distress to central autonomic networks that overlap with the salience network[46, 47] and depression to salience-emotional networks [48]. The interaction between the separate networks may subsequently result in the unified tinnitus percept that may vary depending on changes of the within and between network interactions/connectivity, which might explain—at least to some extent—the temporal variability of the tinnitus percept.

### Multifactorial Aetiology

Our knowledge about etiological factors and the mechanisms by which they cause tinnitus has increased considerably over the last decades. However, many aspects are still unknown, especially the question of what exactly triggers the onset of tinnitus in a particular patient. To put it in another way, why does hearing loss lead to tinnitus in some patients and not in others? Many people suffer from hearing loss, and possibly a second factor may be essential as alluded to before: somatosensory, visual or vestibular modulation.

Yet, other risk factors exist, such as stress, whether psychological or physical stressors, that may be involved in the generation and maintenance of tinnitus [49, 50]. Also trauma to the auditory system is a risk factor, whether a physical trauma [24], noise exposure [51–53], drugs (tobacco [54], cannabis [55], heavy alcohol [16]) or medication (antibiotics, antitumor agents, NSAID, salicylate, antidepressants, ACE inhibitors [56, 57]), and toxins (chromium, cadmium, manganese)[58].

Thus, tinnitus may result from a multifactorial process, a consequence from multiple small accumulating risk factors. Clinically, we indeed often find combinations of such risk factors in individuals with tinnitus and assume that the various risks may accumulate in individual cases. Nevertheless, it is impossible to predict whether a person with these risk factors develops tinnitus or not, if and when the person develops tinnitus, and how burdensome the tinnitus will be.

### Relevance of Aetiological Factors for Tinnitus Management

In the clinical management of tinnitus patients, one should try to identify and modify the risk factors that may have contributed to the onset of tinnitus. In rare cases, the successful treatment of the risk factor may cure tinnitus (e.g. in patients receiving a cochlear implant in case of deafness [59, 60]). In many other cases, tinnitus burden can be lowered by specific interventions (e.g. hearing aids that compensate for the hearing loss[61]); in some cases, the risk factor treatment has a clear beneficial effect only during the acute phase and sometimes it has no effect at all. These observations suggest that there may be additional, so far unknown factors that play a role in the development of tinnitus and that mechanisms involved in the maintenance of tinnitus may differ from the mechanisms relevant for the onset. The natural history of tinnitus does show that once tinnitus is present it is likely to remain in 80% of patients, with 20% of patients having a complete spontaneous resolution within 4 years [62]. Among those who still have tinnitus, 10% worsen, 10% improve and 80% remain unchanged [62]. This suggests that the tinnitus generating network may change over time [63], potentially making it more difficult to alter when it has become chronic. It has indeed been suggested that once the tinnitus is present for 4 years it may become more difficult to treat [64–66]. Hypothetically, this is assumed to be related to the fact that the tinnitus becomes linked to the self-perceptual default mode network, i.e. the tinnitus becomes part of one’s self-identity [42].

## Perceptual Variability

### Tonal or Noise-Like Tinnitus

There is a high perceptual variability of tinnitus. Tinnitus can be perceived as whistling, buzzing, chirping, hissing, humming, roaring, or even shrieking, with the same tone or with different tones or a combination of tones and noise. It has been hypothesised that tonal tinnitus is related to increased activity in the classical pathways (also known as lemniscal pathways) and the noise-like tinnitus is related to increased activation in the non-classical (extralemniscal) pathways [67, 68]. While the classical pathways encode the tinnitus frequency accurately, the extralemniscal pathways process the signal faster, but less accurately and are closely connected with non-auditory areas, e.g. the amygdala or the insula [69]. However, there is little experimental evidence to support this hypothesis [67, 70, 71].

### Tinnitus Localisation

Tinnitus can be perceived unilaterally (about 45%), bilaterally (about 45%) or non-lateralised (about 10%) [72]. This variability can at least be partially explained in case of unilateral hearing loss. In this case, tinnitus is typically on the side of the hearing loss, which supports the theory of deafferentiation. In many cases, however, it remains unclear what predicts tinnitus localisation, especially in patients who experience the tinnitus inside their head. Moreover, some patients have difficulties localising their tinnitus or report that the localisation changes over time. Furthermore, several studies have demonstrated that the perceived tinnitus pitch corresponds to the area of the most pronounced hearing loss [17, 18]. This finding supports the theory of tinnitus as a phantom sound, resulting from the brain’s effort to compensate for the lack of auditory input [73]. Moreover, it suggests that tinnitus generation is mediated by a lack of feed forward inhibition, as a lack of lateral inhibition would result in a tinnitus pitch at the edge frequency between normal hearing and hearing loss [74].

### Pulsatile Tinnitus

A pulsatile, pulse-synchronous tinnitus character is suggestive of abnormal blood flow, either due to a vascular abnormality (e.g. stenosis, av-fistula or malformation) or by increased blood flow (e.g. anaemia) or, less commonly, a microvascular conflict [75, 76]. However, in many patients who report pulse synchronous pulsatile tinnitus, no such abnormalities can be detected. A possible explanation is that these people might be particularly sensitive to body sounds and therefore even perceive sounds generated by regular blood flow and consequently transmitted to the inner ear via bone conduction [77]. Moreover, the transmission of body sounds to the cochlea could be facilitated by increased intracranial pressure [78].

### Variability of Tinnitus over Time

Some patients mention that tinnitus changes pitch and intensity over time which can be traced by a tinnitus tracker that demonstrate these changes may result from behavioural and emotional factors during the same or the previous day [79]. Also, other forms of temporal variability have been reported, e.g. related to sleep. Patients displaying sleep-modulated tinnitus have deteriorated sleep quality, and the tinnitus changes may be related to REM-sleep impairment [80]. Not uncommonly a regular 3- or 4-day sawtooth pattern is expressed, in which 2 bad days are systematically followed by one good day without obvious influencing factors. Up to now, no satisfying explanation for these latter phenomena has been proposed.

## Comorbidities

### Comorbidities: Risk Factor or a Consequence of Tinnitus

Tinnitus is associated with many comorbidities. These comorbidities in turn are an important aspect of tinnitus as they are of great importance for the individual burden of disease [81], but are also important as starting point for the therapeutic management. Some of them may represent risk factors (e.g. hearing loss), others may be consequences (e.g. difficulties concentrating) and some may be both (e.g. depression or anxiety). It is not always possible to distinguish the extent to which a comorbidity is a risk factor or a consequence of tinnitus, but regardless, there is likely to be an overlap of pathophysiological mechanisms. For example, insomnia is the most distressing comorbidity for many (60%) tinnitus patients [82, 83] and there is much overlap in the mechanisms of psychophysiological insomnia and tinnitus [9]. Moreover, tinnitus-associated emotional and cognitive distress and somatic complaints relate to insomnia [84, 85].

### Hyperacusis, Misophonia and Phonophobia

The frequent combination of tinnitus with hyperacusis [86, 87] suggests that both disorders may be related by an increased gain in central auditory pathways [88]. In the case of misophonia, the common pathway might be the mechanism, which generates the aversive character of specific sounds [89, 90], whereas in phonophobia, fear of loud sounds is the common denominator [90]. Some, but not all, studies [91, 92] suggest an increased risk of arterial hypertension among tinnitus patients, which might be an indicator of increased sympathetic activation, which is in keeping with stress as modulating tinnitus [46, 50, 93].

### Tinnitus and Pain

Tinnitus is also related to various headache syndromes [26, 94], as well as temporomandibular pain and cervical pain [95], but also fibromyalgia [27]. Comorbidity with pain syndromes can be explained by shared peripheral (C2 and trigeminal nerve) [96, 97] and central mechanisms of chronic pain and chronic tinnitus [3, 11, 98–101].

## Amount of Tinnitus Suffering

Tinnitus with associated suffering is defined as tinnitus disorder [99], i.e. a pathology in its own right, and not a symptom associated with another pathology. The extent to which tinnitus affects or distresses varies from person to person. Most people with tinnitus are not severely impaired by their tinnitus [102]. On the other hand, 20% of people with tinnitus are severely disturbed in all aspects of their life and may even be suicidal [103]. Knowledge about the factors that determine tinnitus severity is incomplete. Age, hearing difficulties, sleep problems, work noise exposure, ototoxic medication, and neuroticism determine whether someone develops bothersome tinnitus or not [62, 85]. People with high scores on neuroticism scales are more severely impaired [104–107], and sex also plays a role with women being more likely to suffer from severe tinnitus [12, 85]. Perceptual characteristics of the tinnitus gestalt, such as tinnitus loudness or tinnitus pitch, appear to play a role as well [108–110]. However, all the factors mentioned explain only to a small extent the degree of tinnitus suffering. From a neurobiological perspective, it is assumed that high level of distress and high level of tinnitus burden are reflected by a co-activation of stress-related brain networks [3], but here too it remains unclear what determines whether these networks are co-activated or not.

## Tinnitus Modifying Factors

### Tinnitus Modification by Sounds

In the vast majority of patients, tinnitus is modified by various factors. Apart from stress and emotions modifying tinnitus [111], the change caused by external sounds is the most investigated. Sounds can mask the tinnitus and the masking effect can outlast the stimulation period. This phenomenon is known as residual inhibition, varies from patient to patient and depends on the type of sound [112–114]. In general, sounds similar to the tinnitus sound are more efficient for masking and triggering a silent period [112–114]. These effects can be well explained by the concept that tinnitus is an expression of central disinhibition resulting from reduced feed forward inhibition because of reduced auditory input. An increase in input, in turn, leads to an increase of feed forward inhibition and reduces the tinnitus perception. However, sounds do not always reduce tinnitus. Many patients report that certain sounds increase their tinnitus. Whether a sound reduces or increases an individual’s tinnitus may depend on the type of the sound and its loudness and may vary from person to person.

### Tinnitus Modification by Head, Neck or Face Movements

Similarly, manoeuvres of the head, neck or face can modulate the tinnitus percept in many people with tinnitus. This phenomenon is considered as expression of the interaction between the somatosensory and the auditory system [7].

### Tinnitus Modification by Attention and Stress

Tinnitus perception can also be reduced by distraction. Most patients with tinnitus report that the tinnitus fades into the background, when they focus their attention on activity. Imaging studies have shown that tinnitus reduction via distraction is related to reduced neuronal activity in the auditory cortex [115]. Some patients report that the intensity of tinnitus increases in stressful situations [49, 93]. This can be explained by a general enhancement of alertness to sensory signals under sympathetic activation. However, not all persons experience the same situation in the same way. A recent example for this was the COVID-19 pandemic where some tinnitus patients perceived the situation as stressful and described an increasing tinnitus, while others perceived less stress during the pandemic and reported a decrease of tinnitus loudness [106].

### Tinnitus Modification by Sleep

Less understood is the modulating effect of sleep [84]. Many tinnitus patients report that their tinnitus during the day depends on the quality of their sleep the night before. The better the sleep at night, the lower the intensity of the tinnitus [80]. In contrast, napping during the day exacerbates tinnitus in a subgroup of patients. The mechanisms underlying these observations are still unclear and may be elucidated by systematic polysomnography studies in these patients [116].

## Response to Treatment

### Heterogeneity in Response to Treatments

Many different treatments have been investigated for tinnitus [117]. Most treatment studies found no significant difference between the intervention group and the control group and were therefore considered negative. In most of these studies, however, the response to treatment was highly heterogeneous. This means that some study participants responded positively to the intervention, even though most others showed a negative response. It is obvious that it would be highly desirable to identify distinct tinnitus subtypes that respond well to specific treatments or at least to find reliable criteria that can predict the outcome of a specific intervention. A first step in this direction was made by analysing self-reported effects of various therapeutic interventions in a large sample. This study has shown that response to certain treatments predicts the outcome of other treatments [118]. This result is the proof that there exist tinnitus subtypes that differ in their response to specific treatments. This also means that treatment response can be improved by patient subtyping. Based on these findings, decision support systems are currently being developed to help clinicians to select treatment based on individual patient characteristics [15].

### Counselling and Cognitive Behavioural Therapy

The results from specific treatments also provide clues for a better understanding of the pathophysiology of tinnitus. Most established in tinnitus treatment are counselling and cognitive behavioural therapy. It is likely that both methods work via similar mechanisms, namely by improving cognitive control and reducing dysfunctional behaviours. This in turn indicates that neuronal activity and connectivity in stress- and anxiety-related networks in tinnitus patients can be modulated by cognitive control and unlearned by behavioural training.

### Hearing Aids and Cochlear Implants

In tinnitus patients with hearing loss, a therapeutic improvement of hearing also leads to tinnitus reduction, both by hearing aids [61] and cochlear implants [119, 120]. This finding clearly supports the concept that tinnitus results from the brain’s effort to compensate for the reduced auditory input. The effect is most pronounced with cochlear implants [59], which can be explained by the fact that cochlear implants provide input to deafferentiated auditory nerve fibres. Other forms of hearing improvement, such as middle ear surgery or hearing aids, improve hearing by increasing sound pressure levels in the inner ear, but cannot reactivate deafferentiated neurons. Accordingly, their effect on tinnitus is significantly less pronounced compared to cochlea implants [121].

### Pharmacological Treatment

The transient suppression of tinnitus after intravenous lidocaine suggests that tinnitus can be effectively addressed pharmacologically [122]. Unfortunately, the side effects of lidocaine do not allow its long-term use and no other drug has shown comparable efficacy. In cases where no effective drug targets have been identified in clinical trials, studies in animal models of tinnitus suggest the involvement of potassium channels and the GABA-ergic as well the glutamatergic system [123]. Some insights into the molecular mechanisms of tinnitus also come from the information collected on drugs, which may induce tinnitus as side effects [56].

### Brain Stimulation and Neuromodulation

Various forms of brain stimulation have been tested and have demonstrated that stimulation of single targets (e.g. the auditory cortex or the dorsolateral prefrontal cortex or the anterior cingulate) has rather limited efficacy [124]. More promising results are provided from recent efforts with bimodal stimulation, consisting of a combination of sound stimulation and electrical stimulation of somatosensory afferents. This approach has shown considerable improvements of tinnitus in several studies [125–128]. These findings can be interpreted as indirect support for the idea that tinnitus is related to abnormalities in different interacting brain networks [6, 42]. In similar vein, it can be considered that a pharmacological approach that considers a cocktail of medications targeting multiple different neurotransmitter receptors and/or ion channels [129] may be the analogue of multimodal neuromodulation.

## Conclusion

Many clinical aspects of tinnitus are still incompletely understood and cannot be fully explained by current pathophysiological models. Further research should focus on a precise clinical characterisation of these aspects and on their neurophysiological basis to gain more detailed insights into the pathophysiology of the different tinnitus subtypes as well as on potential treatment targets. This might help to elucidate whether there exists a final common pathway of the various forms of tinnitus that can be therapeutically targeted or whether different treatment approaches for various subtypes are the more promising approach.

Considering that single pharmacological and single anatomical targets do only yield limited beneficial effects for tinnitus, it can be conceived that pharmacological strategies focussing on multiple targets or multimodal neuromodulation approaches or a combination of medication, neuromodulation and auditory approaches may be superior treatment approaches. The superiority of such a combinational approach may either be based on synergistic action or simply on the shotgun principle, as the combination of several therapies increases the chance that the right therapy is included.
